# Concentration

**Published:** 1862-06

**Authors:** 


### Editorial.

### CONCENTRATION.

In the execution of any difficult work, when skill is required, all will admit the fact, that undivided attention or concentration of the mind is necessary.

The public speaker, who will make himself clearly understood, must not only comprehend his subject, but in the deliverance must have his whole attention fixed upon the matter in hand. The same is true in the performance of any operation where skill and delicacy of manipulation are required; in commanding and guiding the fingers to a delicacy of touch and execution in the performance of that which is almost always regarded by the patient as rough and disagreeable at best. In nothing is this more fully realized than in the operation of filling teeth; and especially in difficult cases, when oftentimes such a diversion or division of the attention results in the ruin, or at least great injury of the operation. Hence, we deem it important that a dentist should be spoken to as seldom as possible during the performance of his operations, and especially those attended with difficulty.

A tooth is a delicate organ, and when operated upon should be touched lightly and skillfully; by inattentive cutting and thrusting a part may be cut away that should remain, or a frail part broken down, or a sensitive part unnecessarily perforated, or rudely cut.

Then in introducing fillings, carelessness or inattention is productive of a multitude of evils; among which is an imperfect introduction of the gold; a want of perfect adaptation and consolidation of the filling; an unequal distribution of the force employed; slipping of the instrument, and thereby wounding or lacerating the soft parts; and in connection with this, an inattentive operator will generally have a restless patient.

All are not alike easily diverted from their work; but usually direct questions asked during manipulation is productive of pernicious diversion ; or a conversation carried on by other parties in presence of the dentist will have the same effect.

How often have we been wholly involved, both mentally and physically, in an operation—all concentration and fixed attention, to make an adjustment, secure a point, and attain an object that is almost out of reach ; when just at that point somebody puts a question or asks our opinion about some trivial thing, or starts a vigorous conversation about the war ; (all of which we have wished on "t'other side of Jordan" till our work was complete), when we would just as soon they would come up and "blaze away with a club." It would be but little, if any, more annoying, for we have either to answer the question, and to be interested in the conversation, or appear rude by not replying, which is about equally annoying.

We prefer to have no one, not even an assistant, to speak during a difficult operation, and nothing done that will divide the attention. T.

